# A Novel Cre/lox71-Based System for Inducible Expression of Recombinant Proteins and Genome Editing

**DOI:** 10.3390/cells11142141

**Published:** 2022-07-07

**Authors:** Maxim Karagyaur, Daniyar Dyikanov, Pyotr Tyurin-Kuzmin, Stalik Dzhauari, Mariya Skryabina, Maksim Vigovskiy, Alexandra Primak, Natalia Kalinina, Vsevolod Tkachuk

**Affiliations:** 1 Institute for Regenerative Medicine, Medical Research and Education Center, Lomonosov Moscow State University, 27/10, Lomonosovsky Ave., 119192 Moscow, Russia; vigovskiy_m.a@mail.ru (M.V.); tkachuk@fbm.msu.ru (V.T.); 2 Faculty of Medicine, Lomonosov Moscow State University, 27/1, Lomonosovsky Ave., 119192 Moscow, Russia; danidy@inbox.ru (D.D.); tyurinkuzmin.p@gmail.com (P.T.-K.); stalik.djauari@yandex.ru (S.D.); skrebbka@gmail.com (M.S.); primak.msu@mail.ru (A.P.); n_i_kalinina@mail.ru (N.K.)

**Keywords:** controlled expression, artificial transcription factor, Cre recombinase, gene therapy, functional genomics, genome editing

## Abstract

In this study, we developed a novel Cre/lox71-based system for the controlled transient expression of target genes. We used the bacteriophage P1 Cre recombinase, which harbors a short, highly specific DNA-binding site and does not have endogenous binding sites within mouse or human genomes. Fusing the catalytically inactive form of Cre recombinase and the VP64 transactivation domain (VP16 tetramer), we constructed the artificial transcription factor Cre-VP64. This transcription factor binds to the lox71 sites within the promoter region of the target gene and, therefore, upregulates its expression. We tested the Cre-VP64/lox71 system for the controlled expression of several genes, including growth factors and the genome editor CRISPR/Cas9, and obtained superior efficiency in the regulation of transgene expression, achieving a high expression level upon induction together with low basal activity. This system or its modified forms can be suggested as a novel effective tool for the transitory controlled expression of target genes for functional genomic studies, as well as for gene therapy approaches.

## 1. Introduction

Modern approaches to regenerative medicine demand sophisticated systems for controlled expression of target genes. Currently, several systems for controlled expression are used, induced by doxycycline, tamoxifen or ecdysone. However such expression systems have several limitations, including requirements for the activation of receptor/transcription factor permanent expression [1,2,3] and non-specific activation of some genes [4,5,6,7], as well as undesirable influence on cell metabolism and proliferation [8,9]. Alternatively, several expression systems driven by inducers, which simultaneously act as a transcription factors (e.g., CRISPRa) [10], have been developed. These systems also have some limitations; primarily, hindered delivery due to their large size (about 1600 amino acids) and the requirement that these transcription factors be targeted toward multiple sites within the promoter region of the gene of interest.

To develop an advanced system for controlled transgene expression, we considered several DNA-binding proteins, including transcription factors, that were small (less than 350 aa long), demonstrated high specificity and affinity for their DNA-binding sites and did not have endogenous binding sites within human or mouse genomes (to exclude off-target activation). We found that the bacteriophage P1 Cre recombinase fulfills these requirements. It is 343 aa long, harbors a short, highly specific DNA-binding site and does not have endogenous binding sites within mouse or human genomes [11,12]. Furthermore, a specific modification of this protein (TAT-Cre) strongly facilitates its uptake by target cells in vitro [13].

In this study, we constructed the artificial transcription factor Cre-VP64 using the catalytically inactive form of Cre recombinase and the VP64 transactivation domain (VP16 tetramer). This transcription factor binds to the lox71 site within the promoter region of the target gene and, therefore, upregulates its expression.

We tested this novel Cre-VP64/lox71 system for the controlled expression of several genes, including growth factors and the genome editor CRISPR/Cas9, and we obtained superior efficiency in the regulation of transgene expression, achieving a high expression level combined with low basal activity. This system can be suggested as a novel, effective tool for the transitory controlled expression of target genes for functional genomic studies, as well as for novel gene therapy approaches.

## 2. Materials and Methods

### 2.1. Genetic Constructs Encoding Cre-VP64 Transactivator and lox71-Based Promote

To build an artificial transcription factor we obtained recombinase-deficient isoforms of Cre recombinase, namely dCre (A36V, E129R, K201A), Cre_Δ331_ and dCre_Δ331_ (Δ331, A36V, E129R, K201A), using an LV-Cre pLKO.1 construct containing a P1 Cre recombinase gene (Addgene, Watertown, MA, USA, #25997). These variants were inserted into pVax1 plasmid (ThermoFisher Scientific, Waltham, MA, USA, #V26020) under a constitutive CMV promoter using HindIII-XbaI sites. The S(GGGGS)_3_-linker with VP64 transactivation domain was obtained from the lenti-dCas9-VP64_Blast construct (Addgene, Watertown, MA, USA, #61425) and inserted into pVax1 encoding dCre or dCre_Δ331_ using XbaI-ApaI sites. To promote the nuclear localization of dCre-VP64 variants, an NLS sequence was added using NheI-HindIII restriction sites, and Kozak consensus sequences were added before the NLS start codon.

To create synthetic promoters activated by the Cre-VP64 transcription factor, basal promoter sequences were used, including BREu, TATA, BREd, INR and DPE [14,15]. Three variants of core-promoters (GenScript, Nanjing, China) were built up and inserted into the pVax1 plasmid instead of the CMV promoter, including coreA (A for short) 5′-GAATTCATTACTTACG GTAGATGGCTCGAGCACGCCTATATAAGGTATGTTCGTTTACATACCCTCATTCTGGAGCGTTACATAACTTACGGTAAATGGCCCGC; coreB (B for short) 5′-GAATTCA TTACTTACGGTAGATGGCTCGAGCACGCCTATAAAAGGTATGTTCGTTTACATACCCCCACTTCGGAGCGTTACATAACTTACGGTAGACGTAGACGTAAATGGCCCGC and coreC (C for short) 5′-GAATTCATTACTTACGGTAGATGGCTCGAGGGCGCCTA TATAAGCAGTACTGACTGACTGACCCTCATTCTGGAGACCATCGATGCACTGTTCCGTTACATAACTTACGGTAAATGGCCCGC. Minimal promoter of TRE3G system Tet-On 3G (Takara Bio, Kusatsu, Japan, #631168), coreD (D for short) 5′-GAATTCTTTAGACGCGTACGGTGG GCGCCTATAAAAGCAGAGCTCGTTTAGTGAACCGTCAGATCGCCTGGAGCAATTCCACAACACTTTTGTCTTATACCAACTTTCCGTACCACTTCCTACCCTCGTAAA was used as a reference to assess the efficiency of A/B/C minimal synthetic promoters. One, three or six lox71 (5′- TACCGTTCGTATAGCATACATTATACGAAGTTAT) Cre-VP64 binding sites [16], separated by spacer 5′-GAATTGAGCACTAG, were inserted before the A/B/C/D minimal promoters.

Sequences encoding GFP, cAMP sensor PKA-spark [17], brain-derived neurotrophic factor (BDNF) from pNCure plasmid [18] or Cas9 nuclease were placed under Cre-VP64-regulated promoters to analyze the efficiency of the novel expression system. All constructs were verified using Sanger sequencing.

### 2.2. Cell Culture and Transfection

The Cre-VP64 expression system was tested on a HEK293T cell line (ATCC, Manassas, VA, USA, #CRL-3216™). Cells were cultured in Dulbecco’s Modified Eagle Medium (DMEM), high glucose, supplemented with 10% fetal bovine serum (FBS) and 100 U/mL of penicillin/streptomycin (all from Gibco, Waltham, MA, USA). The medium was changed every 3–4 days. Cells were transfected with PEI 25 kDa linear (Polysciences, Warrington, PA, USA, #23966-1) according to a previously published protocol [19]: 2 μg of plasmid DNA per 1 mL of culture medium, NP ratio ~30. To normalize the dosages of reporter (GFP) or transgene (PKA-spark, BDNF, Cas9) plasmids, their amounts were limited to 50% (or 1 μg) within the transfection mixture, with the other 50% (1 μg) consisting of the inductor-encoding plasmid or the “ballast” empty pVax1 vector (for the negative control group).

### 2.3. Inducible Transgene Expression Using Cre-VP64/lox71 System

To evaluate the efficiency of the Cre-VP64/lox71 expression system components, HEK293T cells were transfected with a plasmid encoding a reporter gene (GFP) under the control of one of the lox71 promoters (lox71_1/3/6_-A/B/C/D) and combined with either a plasmid encoding an inductor (one of the P1 Cre recombinase isoforms) under the control of the constitutive CMV promoter or with the empty pVax1 backbone (to assess the basal lox71 promoter activity) in a 1:1 molar ratio. In the positive control groups, cells were transfected with pVax1-CMV-GFP combined with the empty pVax1 vector or pTRE3G-GFP combined with the pCMV-Tet3G vector (Takara Bio, Kusatsu, Japan, #631168) or the empty pVax1 backbone (to assess the basal TRE3G promoter activity) with a 1:1 molar ratio. To assess cell basal autofluorescence, HEK293T cells were transfected with the empty pVax1 plasmid (HEK293T in figures).

To confirm the ability of the Cre-VP64/lox71 system to promote transgene expression, HEK293T cells were transfected with pVax1 plasmids encoding a PKA-spark cAMP sensor or BDNF under the control of lox71_3_-C or lox71_6_-C promoters and combined with the pVax1-CMV-dCre_Δ331_-VP64 vector or the empty pVax1 backbone (to assess the basal lox71 promoter activity) in a 1:1 molar ratio. All of the groups above were reproduced at least in triplicate.

HEK293T cells were transfected as described above. Cells transfected with the components of the pTRE3G/pCMV-Tet3G doxycycline-induced system were given doxycycline 6 hrs after the transfection and the final concentration of doxycycline 1 μg/mL in the culture medium was maintained for 42 hrs. Transgene expression was assessed 48 hrs after the transfection. The expression of fluorescent proteins, such as GFP or PKA-spark, was assessed using flow cytometer BD LSRFortessa™ Cell Analyzer (BD, Franklin Lakes, NJ, USA). The gating strategy can be found in Supplementary Figure S1. Cells with fluorescence intensity greater than 700 units (FITC-A) were counted as GFP-positive cells. Cells with fluorescence intensity greater than 20,000 units (FITC-A) were count as highly GFP-positive cells. At least 10,000 cells were analyzed for each sample; all samples were in triplicates. The median fluorescence intensity (MFI) was measured for the total GFP-positive population.

BDNF concentration in the culture medium was analyzed using a Human Free BDNF Quantikine ELISA Kit (R&D Systems, Minneapolis, MN, USA, #DBD00), according to the manufacturer protocol.

The multiplicity of transgene (GFP, PKA-spark or BDNF) expression was also analyzed with Real-Time PCR using qPCRmix-HS SYBR + LowROX (Evrogen, Russia, #PK156L). The following primers were used: 5′-CCCGACAACCACTACCTGAG and 5′- GTCCATGCCGAGAGTGATCC (Tm = 59 °C, 117 bp) for GFP and PKA-spark; 5′-GGCGGCAGACAAAAAGACTG and 5′-CACTGGGAGTTCCAATGCCT (Tm = 59 °C, 177 bp) for BDNF. Transgene expression was normalized to beta-actin, 5′-TGGCATCCACGAAACTACCT and 5′- ATCTTCATTGTGCTGGGTGC (Tm = 59 °C, 165 bp).

To increase dCre_Δ331_-VP64 transactivator uptake by the target cells in a peptide form directly from the culture medium, we created a plasmid encoding TAT-dCre_Δ331_-VP64. HEK293T cells were transfected with pVax1-TAT-dCre_Δ331_-VP64, and culture medium containing TAT-dCre_Δ331_-VP64 was collected and applied to HEK293T cells expressing pVax1-lox71_3_-C-GFP in the presence of protamine sulfate (50 μg/mL). The expression of GFP was assessed with flow cytometry as described above.

### 2.4. Inducible Genome Editing Using Cre-VP64/lox71 System

To analyze the feasibility of the novel Cre-VP64/lox71 system for inducible CRISPR/Cas9 genome editing, we modified the plasmid pX458 (Addgene, Watertown, MA, USA, #48138) by replacing the EF1a promoter with the lox71_3_-C core promoter using KpnI-XbaI restriction sites. The expression cassette of a gRNA under the control of U6 promoter was inserted into the plasmid encoding dCre_Δ331_-VP64. We edited the TP53 gene [20] with the previously validated gRNA (5′-TCCTCAGCATCTTATCCGAG), which was selected according to previously published criteria [21]. HEK293T cells were transfected with pX458-lox71_3_-C-Cas9 and pVax1-dCre_Δ331_-VP64-gRNA plasmids using PEI, as described above. Cells transfected with the plasmid pVax1-dCre_Δ331_-VP64 without gRNA were used as a negative control. Since Cas9 expression starts simultaneously with GFP, we selected cells with high GFP/Cas9 expression 48 hrs after transfection using FACS (BD FACSAria™ III, BD, Franklin Lakes, NJ, USA). Genome DNA was isolated from modified and control cells 96 h after transfection. The edited DNA region was amplified using the primers 5′-ACTTTGCACATCTCATGGGGTTA and 5′-GGTGTAGACGCCAACTCTCTCTA (Tm = 62 °C, 654 bp) and sequenced using Sanger sequencing (Evrogen, Russia). Sequencing results were analyzed using Tracking of Indels by Decomposition (TIDE) software (https://tide.nki.nl/, accessed on 6 June 2022) [22].

### 2.5. Statistical Analysis

Statistical analysis was performed using SigmaPlot11.0 software (Systat Software, Inc., Chicago, IL, USA). Numerical data were assessed for normality of distribution using the Kolmogorov–Smirnov test. Differences between the treatment and control groups were analyzed using Student’s t-test or analysis of variance (ANOVA) on ranks (Dunn’s test), depending on whether or not the data were normally distributed. Data are expressed as the mean ± standard deviation or the median (25%; 75%), depending on the test used. We considered differences to be significant when *p* < 0.05.

## 3. Results

### 3.1. Characterization of Cre-VP64/lox71 System

Cre recombinase recognizes loxP/lox71 sites and cleaves DNA using the N-terminal domain (amino acids 1–330), and the amino acid residues from 331 to 343 are responsible for the formation of DNA synaptic structures. The recombinase activity of Cre can be switched off by inactivation of catalytic amino acid residues or deletion of residues 331 to 343 of the polypeptide tail without any disturbance to its DNA binding properties [23,24].

**Figure 1:**
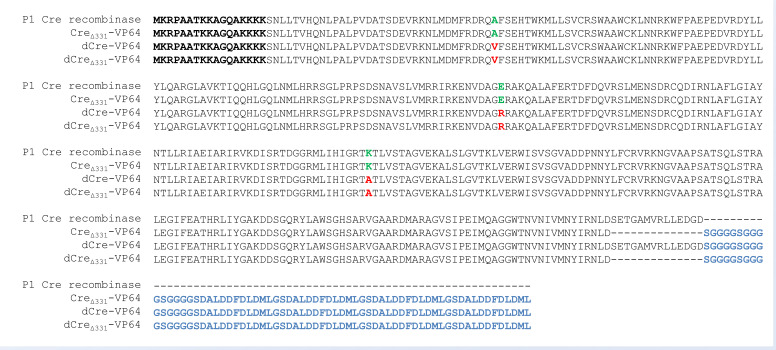
Amino acid sequences of the Cre-VP64 transactivator variants aligned to the sequence of the native P1 Cre recombinase. The NLS sequence is highlighted in bold. The VP64 transactivation domain with the S(GGGGS)_3_ linker is highlighted in blue. Amino acid substitutions in catalytically and synaptically deficient mutant variants are highlighted in red, and the corresponding amino acids in the native sequence are highlighted in green.

Using these data, we developed Cre variants—dCre(A36V, E129R, K201A), Cre_Δ331_ and dCre_Δ331_ (Δ331, A36V, E129R, K201A)—capable of binding to the lox71 sequence but which were at the same time synapsis- and/or catalytic-deficient. The VP64 transactivation domain was added to these deficient Cre variants to create synthetic transcription factors (Figure 1).

**Figure 2:**
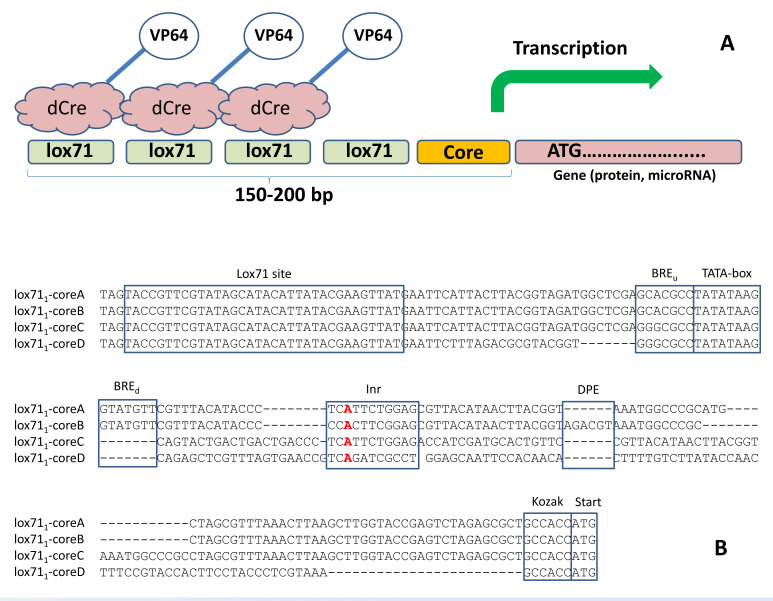
The design of the minimal promoters. (**A**) The scheme for the dCre-VP64 interaction with the core promoter. The positions of multiple lox71 sites are shown. (**B**) The sequences of the developed promoters, which bind the dCre-VP64 transactivator. The transcription initiation site is highlighted in red.

In this study, we developed three minimal promoters composed of the basic promoter components (BREu, TATA, BREd, INR, and DPE) [14,15] and regulatory elements (see Figure 2B for the sequences and the Materials and Methods section for the design details). Their properties, such as basal activity and susceptibility to induction, were largely determined by their primary sequences. To achieve the efficient binding of dCre-VP64 variants to artificial promoters, we inserted one, three or six repeated lox71 sites in close proximity to the core minimal promoter (Figure 2A).

**Figure 3:**
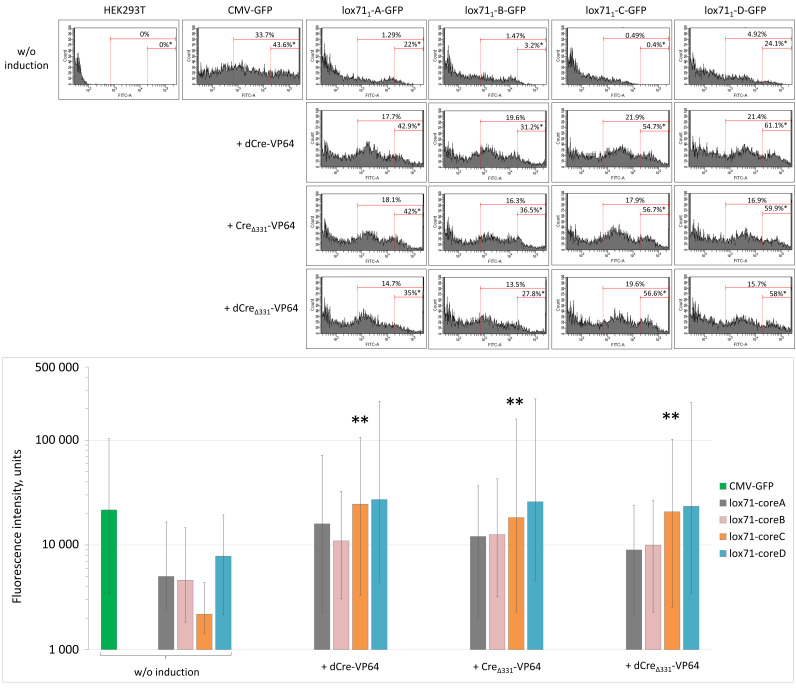
Flow cytometry analysis of GFP expression in HEK293T cells transfected with various combinations of Cre-VP4/lox71 system components. *, The percentage of bright HEK293T cells among all GFP-positive cells; **, *p* < 0.05 compared to GFP fluorescence intensity of cells transfected with pVax1-lox71_1_-coreC-GFP plasmid without induction, *n* ≥ 30,000. In the diagram, data are presented as the median (25%; 75%).

To select the most efficient variants of the Cre-VP64/lox71 expression system, we transfected HEK293T cells with plasmids encoding one of the minimal promoters with or without one of the dCre-VP64 transactivator variants. The lowest background activity and the best signal-to-noise ratio were registered in cells expressing the minimal core promoter C: the median fluorescence intensity (MFI) was 2183 (1429; 4382) units with 0.4% GFP-bright cells before induction and more than 18,247 (2286; 159,304) units and 54.7% GFP-bright cells after induction (*p* < 0.05; *n* ≥ 30,000) (Figure 3); therefore, these cells were selected for further experiments. Next, we compared the ability of dCre-VP64 variants to induce GFP expression from those promoters. All the developed transactivator isoforms—dCre-VP64, Cre_Δ331_-VP64 and dCre_Δ331_-VP64—had almost equal abilities to induce GFP expression and no statistically significant differences were found between them, with an MFI of 24,549 (3287; 106,459) units for dCre-VP64, 18,247 (2286; 159,304) units for Cre_Δ331_-VP64 and 20,759 (2519; 101,943) units for dCre_Δ331_-VP64 (*n* ≥ 30,000) (Figure 3). In view of this, the truncated and catalytically inactive isoform dCre_Δ331_-VP64 was chosen for further experiments: it was shorter than dCre-VP64 (full length, mutated) and highly guaranteed against the undesirable recombinase activity. Thus, we selected a combination of plasmids encoding a minimal core promoter C and the dCre_Δ331_-VP64 transactivator.

**Figure 4:**
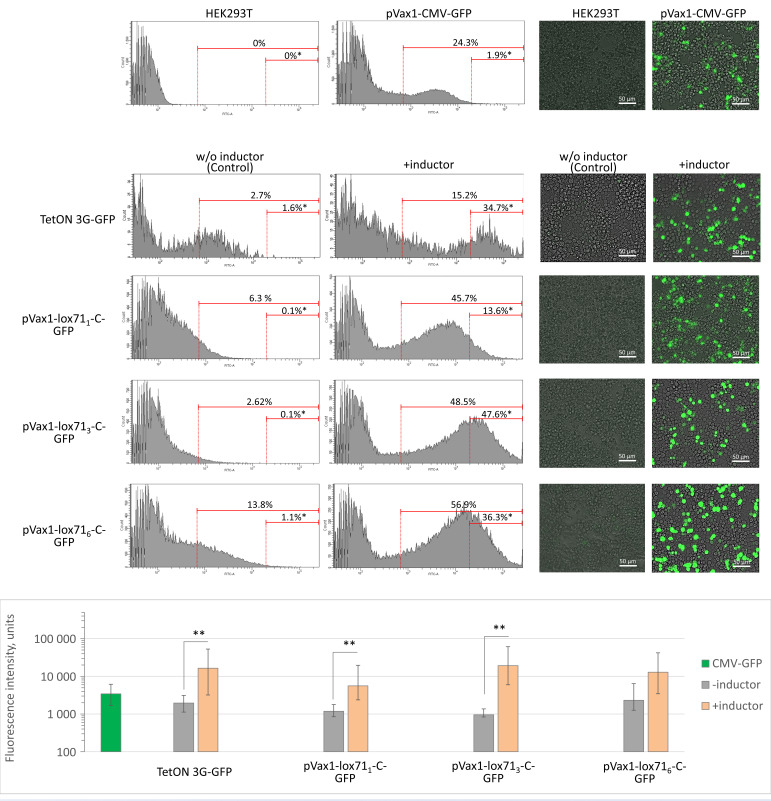
The effect of the number of lox71 sites on the transgene expression. Left panel—flow cytometry analysis of HEK293T cells transfected with the pVax1-lox71-C-GFP with one, three or six lox71 sites or control plasmids 48 h after the transfection. Right panel—fluorescent microscopy of transfected cells 48 h after the transfection. The number of lox71 sites is indicated within the construct name. Inductor: transfection by dCre_Δ331_-VP4 gene for lox71 system and doxycycline for TRE3G promoter. *, The percentage of bright HEK293T cells among all GFP-positive cells; **, *p* < 0.05 compared to GFP fluorescence intensity of cells transfected with the appropriate plasmids without induction, *n* ≥ 30,000. Inductors: doxycycline for TetON 3G-GFP and dCre_Δ331_-VP4 for lox71_1/3/6_-containing plasmids. In the diagram, data are presented as the median (25%; 75%).

Since the efficiency of an inducible expression system strongly depends on the number of transactivator binding sites, we optimized the number of lox71 sites in plasmids encoding minimal promoter variants. HEK293T cells were transfected with plasmids encoding GFP under the control of the lox71 promoter with one, three or six lox71 sites. Basal promoter activity and the magnitude of induction were assessed as GFP fluorescence measured by flow cytometry (Figure 4). The screening of promoter variant efficiencies revealed that the C core promoter variant combined with three lox71 sites had the lowest background activity and the best signal-to-noise induction ratio: the MFI was 951 (832; 1367) units with 0.1% GFP-bright cells before induction and more than 19,178 (5971; 61,059) units and 47.6% GFP-bright cells after induction (*p* < 0.05; *n* ≥ 30,000) (Figure 4). The lox71_1_-C and lox71_6_-C promoters also effectively induced GFP expression; however, they had higher background activity and, hence, lower inducibility.

Thus, the MFI of cells transfected with pVax1-lox71_1_-C-GFP and pVax1-lox71_6_-C-GFP increased after the induction by ~5- and ~5.6-fold, respectively, while the MFI of cells transfected with pVax1-lox71_3_-C-GFP increased after the induction by ~20 (*p* < 0.05, *n* ≥ 30,000) (Figure 4). These data correlate well with the GFP mRNA content. Thus, in HEK293T cells transfected with the genetic construct pVax1-lox71_1_-C-GFP, after induction, the level of GFP mRNA increased by 26 ± 11-fold, and in cells transfected with pVax1-lox71_3_-C-GFP or pVax1-lox71_6_-C-GFP, it increased by 819 ± 191- and 128 ± 36-fold, respectively (*n* ≥ 3) (Figure 4). Here and below, real-time PCR data are normalized to the mean levels of the respective mRNAs for the respective lox71 promoters without the addition of the dCre_Δ331_-VP64 transactivator.

This promoter variant also provided a higher fold of transgene (GFP) expression compared to the A and B promoter variants with the same number of lox71 sites (Supplementary Figure S2) and to the constitutive CMV promoter and doxycycline-induced TRE3G promoter. The lox71_3_-D promoter variant, similarly to its ancestor TRE3G, provided a high level of transgene expression but, at the same time, showed prominent basal activity (Supplementary Figure S2). Thus, the introduction of multiple binding sites for the dCre_Δ331_-VP64 transactivator enhanced the activation potential of this system but could also increase its background activity. Therefore, we concluded that three lox71 sites was the optimal number for the binding of the dCre_Δ331_-VP64 transactivator without excessive basal activity. Similar activity as in the Cre/lox71-based induction system was observed in the HeLa cell line, which demonstrated its reliability (Supplementary Figure S3).

### 3.2. Cre-VP64/lox71 System Provides a High Level of Transgene Expression

**Figure 5:**
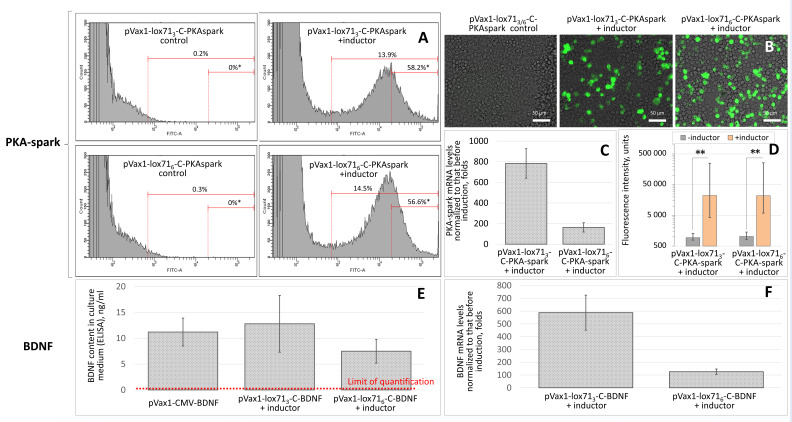
Efficiency of transgene expression in the CreVP64/lox71 system. (**A**) Flow cytometry of cells transfected with constructs encoding PKA-spark. *, The percentage of bright HEK293T cells among all GFP-positive cells. (**B**) Fluorescence microscopy of cells transfected with constructs encoding PKA-spark. (**C**,**F**) Fold enhancement (to “before induction” level) of PKA-spark (**C**) and BDNF (**F**) mRNAs in HEK293T cells 48 h after induction. (**D**) PKA-spark sensor fluorescence intensity of cells transfected with pVax1-lox71_3/6_-C-PKA-spark with or without induction. **, *p* < 0.05, *n* ≥ 30,000. Data are presented as the median (25%; 75%). (**E**) The secretion of human BDNF by transfected cells measured by ELISA 48 h after induction.

To evaluate the system efficiency for a wider target spectrum, we tested the optimized Cre-VP64/lox71 system for inducible expression of cAMP sensor PKA-spark and BDNF. We put PKA-spark or BDNF genes under the C core promoter variant with three or six lox71 sites ahead of it. The MFI of cells transfected with pVax1-lox71_3_-C-PKA-spark (three lox71 sites) and pVax1-lox71_6_-C-PKA-spark (six lox71 sites) increased after the induction by ~24- and ~21-fold, respectively, compared to those without induction (*n* ≥ 3) (Figure 5A).

The level of BDNF expression in the conditioned medium of cells transfected with pVax1-lox71_3_-C-BDNF and pVax1-lox71_6_-C-BDNF (three and six lox71 sites, respectively) after the induction increased by ~1300- and ~750-fold and reached 12.8 ± 5.5 and 7.5 ± 2.3 ng/mL, respectively (*n* ≥ 3) (Figure 5B). The content of human BDNF in the medium of cells transfected with pVax1-lox71_3_-C-BDNF and pVax1-lox71_6_-C-BDNF without induction was below the limit of quantitative determination of the method (~100 pg/mL). However, the expression level of human BDNF by HEK293T cells transfected with the pVax1-hBDNF genetic construct [25], in which BDNF expression is regulated by a strong constitutive eukaryotic CMV promoter, was 11.2 ± 2.7 ng/mL.

The PKA-spark and BDNF expression data obtained by flow cytometry or ELISA also correlated well with the changes in the respective mRNAs. In cells transfected with the genetic constructs pVax1-lox71_3_-C-PKA-spark and pVax1-lox71_6_-C-PKA-spark, the expression level of PKA-spark mRNA 48 h after the induction increased by 783 ± 143- and 163 ± 45-fold, respectively (Figure 5C). In cells transfected with the genetic constructs pVax1-lox71_3_-C-BDNF and pVax1-lox71_6_-C-BDNF, the expression level of BDNF mRNA 48 h after the induction increased by 588 ± 137- and 126 ± 21-fold, respectively (Figure 5F). It was found that, without activation, the lox71_6_-C promoter had ~4.3-fold more prominent basal activity compared to the lox71_3_-C promoter. These data correlate well with the results for the GFP expression, confirming that the promoter variants containing three and six lox71 sites enabled a high level of expression of the target protein, and the highest level of increment in the mRNA and target protein was observed for the promoter variant containing three lox71 sites.

### 3.3. Inducible Genome Editing Using Cre-VP64/lox71 System

**Figure 6:**
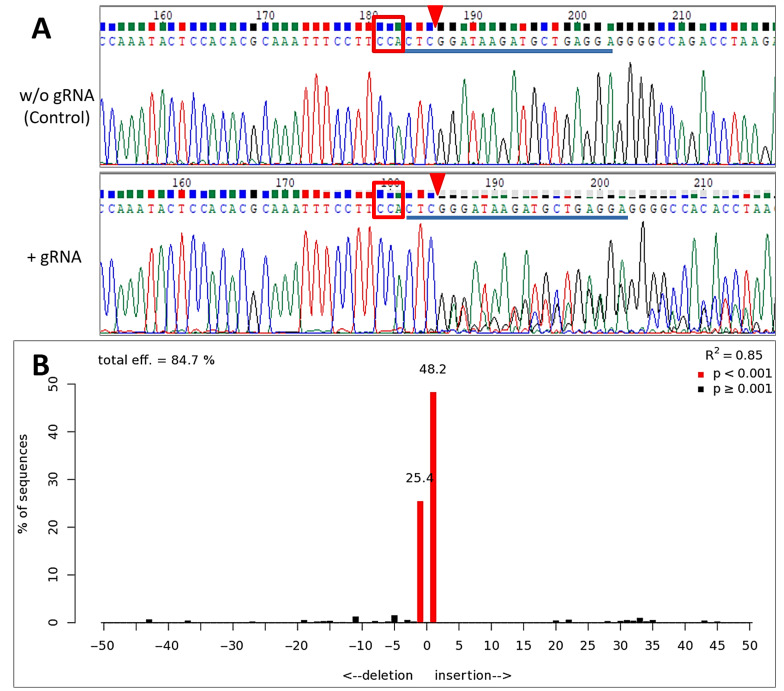
Results of TP53 gene sequencing within the range of genome editing in HEK293T cells after induction of Cas9 programmable nuclease expression. (**A**) An alignment of TP53 allele sequences within the range of genome editing; the blue line labels the gRNA binding region, the red frame marks the PAM sequence and the red triangle marks the Cas9 cleavage region. (**B**) The result from the analysis of the sequencing data in the TIDE program.

The study using HEK293T cell culture demonstrated that the Cre-VP64/lox71 system can be used for inducible genome editing. Thus, in the HEK293T culture transfected with pX458-lox71_3_-C-Cas9 and pVax1-dCre_Δ331_-VP64-gRNA plasmids and enriched in GFP, almost complete editing of the target TP53 allele was observed: one third of the alleles were edited via a deletion of a single nucleotide and two thirds via an insertion of a single nucleotide, which was confirmed through the analysis of the sequencing data using TIDE (Figure 6).

## 4. Discussion

In this study, we developed a new system for inducible transgene expression that is feasible to use for the introduction of intracellular molecular markers and sensors (such as GFP or PKA-spark), for the production of secretory proteins and for CRISPR/Cas9 genome editing.

We studied its properties and found the optimal variants of the Cre-VP64 transactivator molecule and the Cre-VP64-dependent lox71 promoter. It is known that the degree of promoter induction in some artificial inducible systems (e.g., CRISPRa and Tet-On 3G) is regulated by the number of activating complexes associated with it. Transcriptional activity reaches its maximum when four or more transactivator molecules are recruited to the promoter region [26,27]. Here, we demonstrated for a number of transgenes that the insertion of additional binding sites for the dCre_Δ331_-VP64 transactivator could increase the efficiency of the inducible system. The introduction of additional lox71 sites (up to three) into the promoter region upstream of the core promoter led to an increase in promoter activity, presumably due to increased recruitment of dCre_Δ331_-VP64 transactivators to the transcription initiation region. Some of the promoter variants (lox71_3_-C) showed low background activity levels and high inducibility of transgene expression (about 500–1000-fold). This system demonstrated lower background expression and higher inducibility compared to commercially used Tet-ON induction systems. Furthermore, the usage of the Cre-VP64/lox71 system makes it possible to avoid doxycyclin-induced side effects.

Further increase of lox71 site numbers within the promoter up to six led to elevated background transgene expression and, therefore, reduced the system inducibility for HEK293T cells. The obtained result can only be attributed to the investigated variants of the promoters, since the diverse combination of the components of the minimal promoter may have other properties.

In this work, we demonstrated that the Cre-VP64/lox71 system can be used for induced genome editing. When examining the allele in the editing region, we found that the original version of the allele was completely eliminated in the target cells. Presumably, this was associated with both the high level of Cas9 nuclease expression and the relatively high susceptibility of HEK cells to genomic editing. We believe that rapid and multifold increases in the expression of genome editors can be highly beneficial in CRISPR screens of cell lines that are difficult to genetically modify, since to date these screens are mostly used only for easily transfected tumor cell lines [28,29]. In this case, the large and difficult-to-deliver genes of the Cas9 programmable nuclease (or its modifications, CRISPRi, CRISPRa, CRISPR-BE, etc.) and a marker (for example, GFP) under the control of a short and highly inducible promoter are embedded in the cell genome, and a guide RNA in combination with a transactivator (in the form of a plasmid, viral particle or protein/RNA mixture) is delivered to target cells as needed. Temporal control of the expression of the Cas9 editor can be achieved through the use of protein forms of the inducer or short-lived forms encoding its genes (plasmids, non-integrating viral particles, self-inactivating viral particles) [30,31]. For some applications (biotechnology production, etc.), the described system may need further optimization. Hybrid systems (e.g., tamoxifen/Cre-VP64) for reversible gene expression could be obtained.

During the study, we attempted to induce transgene expression by introducing the protein form of the transactivator based on the TAT-form of Cre recombinase (TAT-Cre) into the culture medium, which, according to the literature, is capable of penetrating into cells through endocytosis and triggering the recombination process [13,32]. However, no pronounced effect on GFP expression was observed. We suspect that this may have been due to the low concentration of recombinant TAT-dCre_Δ331_-VP64 transactivator in the culture medium of the producer cells and/or the requirement to optimize the conditions of this process or the amino acid sequence of the obtained transactivator TAT-dCre_Δ331_-VP64.

The specificity of the developed induction system requires further investigation, although it is extremely specific according to the literature [33,34], which states that Cre-mediated recombination can be used for creating cellular and animal transgenic models [35,36]. We expect that recent, cautious reports on the possible off-target activity of Cre recombinase [37] will not be an obstacle to the usage of such a system for research or biotechnological purposes since this modification of the Cre/lox71 system is active mainly within the promoter and coding regions of the genome, and for significant activation of gene expression it requires the presence of several closely grouped transactivator binding sites.

The developed expression system can be combined with the use of the classical Cre recombinase, since the lox71 sites within the lox71 promoter can be replaced with hybrid lox66/71, which is highly resistant to cleavage by Cre recombinase [38].

Thus, the developed Cre-VP64/lox71 induction system is characterized by high inducibility and low background activity and does not require constitutive expression of the inducer receptor, and the inducer is a small protein that can be delivered to target cells, both as a protein molecule (potentially) and as an encoding gene. In this work, we did not test the specificity of this modification of Cre recombinase; however, according to the literature, there are no endogenous Cre recombinase binding sites within the mouse and human genomes, which means it can be used for creating cellular and animal models with high efficiency.

Potentially, the developed system can be used for the inducible expression of protein and/or microRNA genes to solve the problems of functional genomics (for example, for signal potentiation when studying the work of weak promoters) and for inducible genome editing in cell lines since it is characterized by low background activity. The Cre-VP64/lox71 system has a strong ability to multiply the expression of target proteins; therefore, it can be used as is or as with modifications for biotechnological production or to increase the efficiency of gene therapy.

## Data Availability

Data is available on request.

## Supplementary Materials

The following supporting information can be downloaded at: https://www.mdpi.com/article/10.3390/cells11142141/s1, Figure S1: Gating strategy used in the study: 1. HEK cells are gated based on size to exclude debris and large cell clumps. 2. Singlets are gated to exclude doublets. 3. Gating of GFP positive cells; Figure S2: The effect of the number of lox71 sites on the transgene expression for A/B/D minimal promoters (dCreΔ331-VP4 transactivator)—flow cytometry analysis of HEK293T cells transfected with the pVax1-lox71-C-GFP with one, three or six lox71 sites or control plasmids 48 h after the transfection. In the diagram, data are presented as the median (25%; 75%); Figure S3: The effect of the number of lox71 sites on the transgene expression for C minimal promoter (dCreΔ331-VP4 transactivator)—flow cytometry analysis of HeLa cells transfected with the pVax1-lox71-C-GFP with one, three or six lox71 sites or control plasmids 48 h after the transfection. In the diagram, data are presented as the median (25%; 75%).
