# Supplementary material for: A Novel Cre/lox71-Based System for Inducible Expression of Recombinant Proteins and Genome Editing
Source: Cells. 2022 Jul 7;11(14):2141. doi: 10.3390/cells11142141 (PMC9324666; doi:10.3390/cells11142141)
Supplement: Supplementary file 1 [file cells-11-02141-s001.zip › cells-1784354-supplementary.pdf]

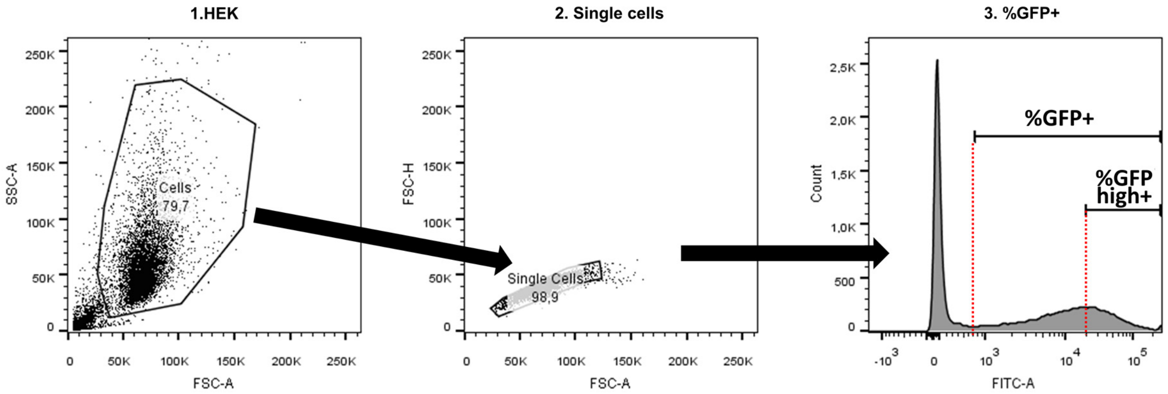


Supplementary Figure S1: Gating strategy used in the study: 1. HEK cells are gated based on size to exclude debris and large cell clumps. 2. Singlets are gated to exclude doublets. 3. Gating of GFP positive cells.

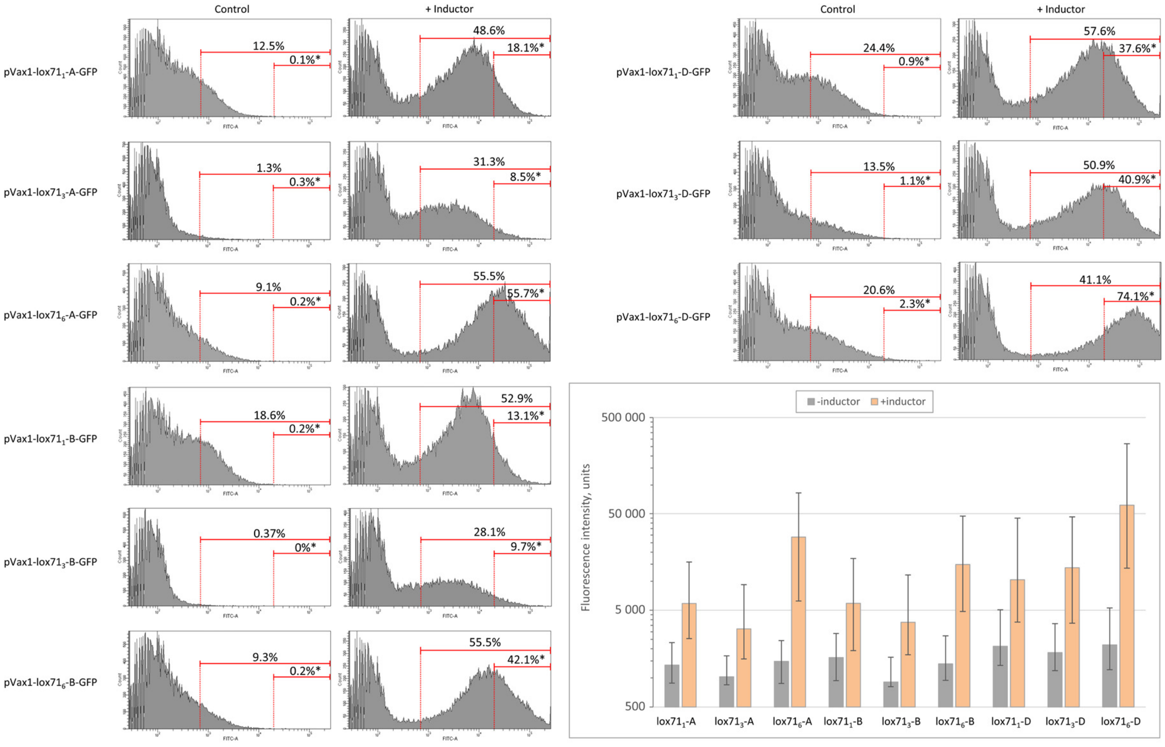


Supplementary Figure S2: The effect of the number of lox71 sites on the transgene expression for A/B/D minimal promoters (dCre$_{\Delta331}$-VP4 transactivator) - flow cytometry analysis of HEK293T cells transfected with the pVax1-lox71-C-GFP with 1, 3 or 6 lox71 sites or control plasmids 48 hours after the transfection. In the diagram data are presented as a median (25%;75%).

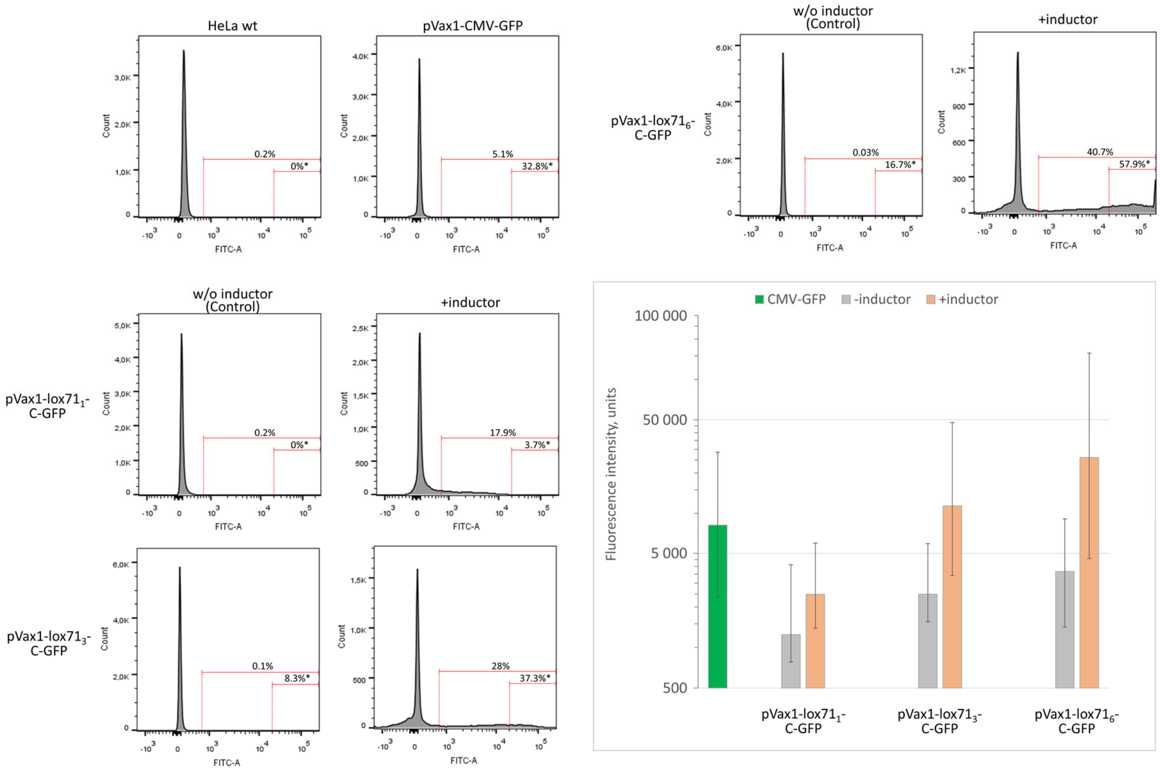


Supplementary Figure S3: The effect of the number of lox71 sites on the transgene expression for C minimal promoter (dCre$_{\Delta 331}$-VP4 transactivator) - flow cytometry analysis of HeLa cells transfected with the pVax1-lox71-C-GFP with 1, 3 or 6 lox71 sites or control plasmids 48 hours after the transfection. In the diagram data are presented as a median (25%;75%).
